# Improved Multi-Stage Rice Above-Ground Biomass Estimation Using Wavelet-Texture-Fused Vegetation Indices from UAV Remote Sensing

**DOI:** 10.3390/plants14182903

**Published:** 2025-09-18

**Authors:** Jinpeng Li, Qiang Cao, Shuaipeng Wang, Jiayi Li, Dongxue Zhao, Shuai Feng, Yingli Cao, Tongyu Xu

**Affiliations:** 1 College of Information and Electrical Engineering, Shenyang Agricultural University, Shenyang 110866, China; ljp@stu.syau.edu.cn (J.L.); caoqiang@stu.syau.edu.cn (Q.C.); 2023240165@stu.syau.edu.cn (S.W.); lijiayi010804@gmail.com (J.L.); sunny_zhao0601@163.com (D.Z.); fengshuai@syau.edu.cn (S.F.); caoyingli@syau.edu.cn (Y.C.); 2 National Digital Agriculture Sub-Center of Innovation (Northeast Region), Shenyang 110866, China; 3 Key Laboratory of Intelligent Agriculture in Liaoning Province, Shenyang 110866, China

**Keywords:** UAV, AGB, multi-feature fusion, discrete wavelet transform (DWT), plant phenotyping, rice

## Abstract

When estimating above-ground biomass (AGB) across multiple growth stages, vegetation indices (VIs) have limitations due to saturation under dense canopies and poor sensitivity to vertically growing organs (e.g., panicles). Discrete wavelet transform (DWT) can extract multi-directional, multi-frequency texture features reflecting canopy structure changes, but its application in crop biomass monitoring is underexplored. Therefore, to evaluate whether DWT-based textures can be used to estimate AGB across multiple growth stages and whether combining VIs can improve estimation accuracy, two-year field experiments involving four rice varieties and five nitrogen treatments were conducted. UAV multispectral images were acquired during the critical growth stages, from which Vis and wavelet textures (WTs) were extracted, and novel wavelet texture indices (WTIs) were constructed. Correlation analysis guided feature selection, and simple regression, multiple linear regression, and Optuna-optimized random forest were employed to develop rice AGB estimation models. The results indicated: (1) Compared to a single WT, the WTIs exhibited higher correlation with rice AGB across different growth stages. (2) Among the three models, the RF model performed best. Specifically, using only VIs to estimate AGB during pre-heading yielded relatively higher accuracy (R^2^ = 0.713), while using WTIs to estimate AGB during post-heading and all-stage yielded higher accuracy (R^2^ = 0.709 and 0.668). (3) Combining WTIs with VIs significantly improves the prediction accuracy of AGB at different growth stages (R^2^ = 0.782, 0.769, and 0.732; RMSE = 114.655, 161.779, and 223.654 g/m^2^), with R^2^ improving by 10–15% and RMSE decreasing by 13–17% compared to the VIs. The study demonstrates that DWT-based textures can effectively assist in the high-precision estimation of rice AGB. Moreover, integrating WTIs with VIs enables accurate and stable prediction of rice AGB under different management practices and varieties, providing an economical and efficient method for estimating rice AGB.

## 1. Introduction

Rice is the main staple for over half of the global population and plays a crucial role in ensuring food security worldwide [1,2]. Above-ground biomass (AGB), a key agronomic parameter that reflects the accumulation and distribution of photosynthetic products in crops, effectively indicates the material accumulation and nutritional status of rice [3]. It has been shown to have significant application value in crop nutritional diagnosis and yield prediction [4,5]. Therefore, achieving rapid, non-destructive, and accurate monitoring of rice AGB at the plot scale is essential for assessing rice growth status, predicting yield, and guiding precision agriculture practices. Currently, the most widely used and accurate method for measuring AGB remains manual sampling and weighing [6]. However, this method is both destructive and time-consuming, making it challenging to meet the demand for large-scale, rapid quantitative monitoring of AGB [7,8].

With the advancement of smart agriculture, low-altitude unmanned aerial vehicle (UAV) platforms present advantages such as flexibility, ease of operation, and low cost, facilitating the acquisition of large-scale, high-spatial-temporal-resolution crop canopy imagery [9,10,11]. Numerous studies have confirmed that UAV platforms equipped with multispectral and visible light sensors exhibit significant potential for monitoring crop growth dynamics, gradually becoming essential technical tools for overseeing the growth of field crops in precision agriculture [12,13,14]. Common methods for estimating AGB using UAV remote sensing imagery primarily include empirical regression models and radiative transfer models [15,16]. Among these, radiative transfer models can simulate the multiple scattering, absorption, and transmission of light within crop canopies, thereby accurately characterizing the influence of canopy structure on light energy distribution [17]. This provides theoretical support and a physical basis for the inversion of crop phenological parameters such as biomass and leaf area index (LAI) [18,19]. However, radiation transfer models typically involve a large number of input parameters during model construction, resulting in complex models with high computational demands that limit their practical application and promotion. In contrast, empirical regression models offer high computational efficiency and simpler implementation and are commonly used to establish quantitative relationships between phenotypic parameters, such as biomass and remote sensing variables.

Among numerous remote sensing variables, VIs constructed based on spectral band combinations can effectively enhance the spectral response of vegetation while minimizing interference from soil background and lighting conditions and have been widely utilized in crop monitoring. Notably, the red and near-infrared bands are highly sensitive to vegetation chlorophyll content, making them effective in characterizing crop growth conditions [20,21]. VIs calculated using these two bands have been widely applied in estimating key physiological and biochemical parameters such as LAI, nitrogen status, vegetation cover, and biomass [22,23,24]. However, due to the large proportion of soil background or elevated canopy biomass, spectral bands or VIs may perform poorly during the early or late stages of crop growth. Some researchers have observed that different VIs exhibit varying sensitivities across different growth stages of crops, leading to recommendations for integrating multiple VIs to enhance monitoring effectiveness and data quality throughout the entire growth stage [9,25]. For rice, its canopy structure undergoes significant changes throughout the growth stage, making it more complex than other vegetation types [26]. During the early stage, low canopy coverage results in canopy reflectance being significantly influenced by the background. As the tillering and jointing stage progresses, an increase in leaf area leads to rapid canopy closure, gradually obscuring the soil background. When the canopy coverage reaches a high level, VIs begin to saturate, resulting in reduced sensitivity and diminished accuracy in reflecting crop growth conditions. After entering the heading stage, rice panicles gradually emerge and are distributed in a disorderly manner, further complicating the canopy structure and spectral characteristics [27,28]. Li et al. [29] used five VIs to estimate biomass at different growth stages of rice, discovering that VIs performed optimally during the jointing stage but exhibited a significant decline in accuracy after the heading stage. Furthermore, Zhou et al. [30] discovered that the accuracy of yield estimation was negatively impacted by rice panicles during the later stages of growth. Consequently, when using remote sensing to estimate biomass during the entire growth cycle of rice, it is essential to take into account the variations in canopy structure at various growth stages and to reasonably choose VIs to facilitate precise estimations. Nevertheless, it is important to emphasize that depending exclusively on the spectral data from remote sensing imagery is insufficient for completely capturing the dynamic changes in canopy structure. This is particularly true in the later growth, when the complexity of the canopy structure increases, severely limiting the accuracy of estimations [31].

Texture analysis is an efficient image processing method commonly used to measure the variability among pixels within a local sliding window in an image [32]. Structural differences in vegetation canopy distribution along vertical and horizontal axes can lead to changes in image texture, providing valuable insights for quantitative assessments of vegetation growth conditions. In recent years, texture features have demonstrated significant application value in estimating crop LAI, AGB, and yield [33,34,35]. Among various texture extraction methods, wavelet analysis has emerged as an effective tool for texture analysis due to its excellent time-frequency localization capability and multi-resolution characteristics, making it widely applicable in feature extraction. Images are decomposed into a low-frequency subband (LL) and three high-frequency subbands (LH, HL, HH) using the discrete wavelet transform (DWT), which, respectively, reflect overall structural information and high-frequency detail changes in different directions [36]. Given that high-frequency texture is typically considered to characterize dense canopy structures, related studies have utilized DWT to extract high-frequency texture for biomass estimation [37,38]. However, high-frequency texture focuses on capturing detailed canopy textures but is susceptible to noise, making it challenging to reliably reflect structural changes in the canopy across different growth stages. Conversely, the low-frequency subband retains the primary structural and brightness information of the image, demonstrating strong noise resistance and providing a more stable reflection of the overall morphological characteristics of crop canopies [18]. However, low-frequency texture is less sensitive to subtle local structural changes and struggles to fully capture differences in canopy texture. Therefore, considering both low-frequency and high-frequency textures during feature construction can theoretically balance the overall morphology and local details of the canopy, thereby enhancing the ability of texture features to characterize structural changes in crop canopies. Drawing inspiration from spectral feature construction methods that enhance sensitivity to canopy spectral and structural characteristics by integrating multispectral information, combining low-frequency and high-frequency wavelet texture features (WTs) to construct wavelet texture indices (WTIs) may further improve the comprehensive representation of both overall and local canopy information. This approach could provide an effective image texture feature construction strategy for monitoring crop growth conditions. Given this, wavelet-based texture measures may serve as an effective indicator for integrating spectral features to improve the estimation accuracy of AGB in rice at different growth stages. However, their effectiveness necessitates further systematic evaluation.

Therefore, in this study, a UAV equipped with multi-spectral sensors was used to acquire multi-spectral images of rice at different growth stages. By calculating VIs and combining them with texture features extracted using DWT, the objectives were: (1) to investigate the relationship between the combination of high-frequency and low-frequency textures extracted from DWT and rice AGB, and to clarify the applicability of different frequency domain features at different growth stages; (2) to assess the impact of spectral and WTs on the estimation accuracy of rice AGB at different growth stages when used individually or in combination with multiple features; (3) to compare the estimation performance of three regression models for rice AGB at different growth stages, identify the optimal model, and analyze and interpret the contributions of various features.

## 2. Materials and Methods

### 2.1. Experiment Location and Design

**Figure 1:**
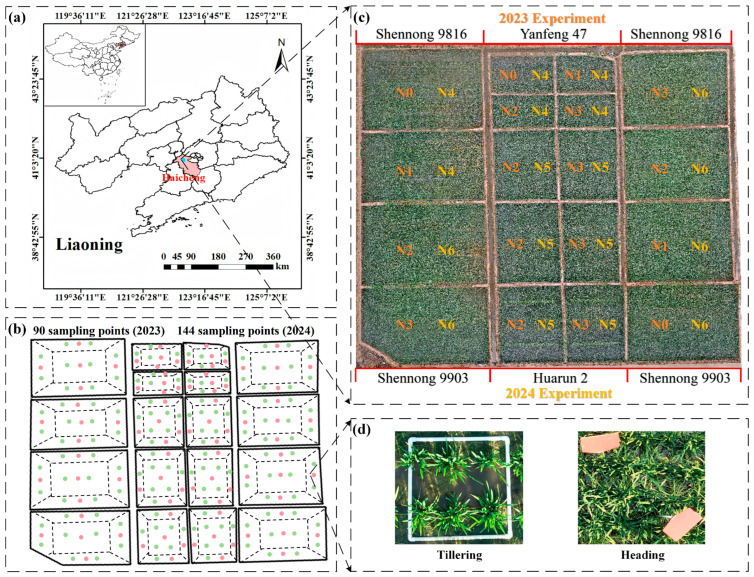
Geographic location of the study area and orthophotography of the field trial sites. (**a**) Field trials were conducted in Haicheng City, Liaoning Province, in 2023 and 2024. (**b**) Sub-maps of sampling points calibrated at individual key growth stages during the two-year trials, with red and green dots representing sampling points from 2023 and 2024 respectively. (**c**) Distribution of different varieties and nitrogen application rates during the two-year trials. (**d**) Sampling point calibration methods at different growth stages (PVC pipes used before canopy closure, and identification tags used for assistance after canopy closure).

In 2023 and 2024, two field trials were carried out at the Precision Agriculture Aviation Research Base of Shenyang Agricultural University, situated in Haicheng City, Anshan City, Liaoning Province, China (Figure 1a, 122°39′18″ E, 40°58′58″ N). This area experiences a temperate continental monsoon climate, characterized by summer temperatures ranging from 20 °C to 28 °C and an annual precipitation average of 721.3 mm, which supports the growth and development of rice. The trials comprised 18 plots featuring various rice cultivars and nitrogen application levels (Figure 1c). A summary of the experiments is provided in Table 1. In 2023, the rice varieties Shenong 9816 and Yanfeng 47 were transplanted on May 28, with four nitrogen application levels (N0 = 0 kg/hm^2^, N1 = 100 kg/hm^2^, N2 = 200 kg/hm^2^, and N3 = 300 kg/hm^2^). The following year, two additional varieties, Huarun 2 and Shen Nong 9903, were transplanted on May 27, utilizing three nitrogen application rates (N4 = 0 kg/hm^2^, N5 = 150 kg/hm^2^, and N6 = 300 kg/hm^2^). Phosphorus and potassium fertilizers were applied at local standard rates of 144 kg/hm^2^ and 192 kg/hm^2^, respectively, while other field management practices adhered to local agricultural standards. Each plot was segmented into five sampling zones, with white PVC pipes marking random 0.5 × 0.5 m^2^ areas for biomass assessment and data collection (Figure 1d). In 2023, there were 90 sampling points established for each trial (Figure 1b, red dots), whereas in 2024, this increased to 144 sampling points (Figure 1b, green dots). As the rice canopy developed and obscured the PVC pipes during the jointing stage, two 2-m-long markers were positioned diagonally opposite the PVC pipes to aid in identifying the sampling zones from that stage onward (Figure 1d).

### 2.2. Rice AGB Data Collection

Destructive sampling of the ground was performed during the crucial growth phase of rice (Table 1). Following the UAV flight, rice plant samples with an area of $0.5\times 0.5\;m^{2}$ were excavated from the designated sampling areas in each experimental field and subsequently transported to the laboratory for rinsing with clean water and root removal. The processed rice samples were placed into paper archive bags labeled with the corresponding sampling areas and then dried in an oven. Initially, the samples were heated to 105 °C for 30 min, followed by a reduction to 80 °C, which was maintained until a stable weight was reached. An electronic scale with a precision of 0.01 g was used to weigh the samples, and the resulting dry weight was converted to AGB per unit area ($g/m^{2}$) according to Equation (1) as follows:

(1) $$AGB_{rice}=\frac{T}{S}$$

where T represents the total weight (g) of AGB in each sampling area, and S represents the sampling area ($0.25\;m^{2}$).

**Figure 2:**
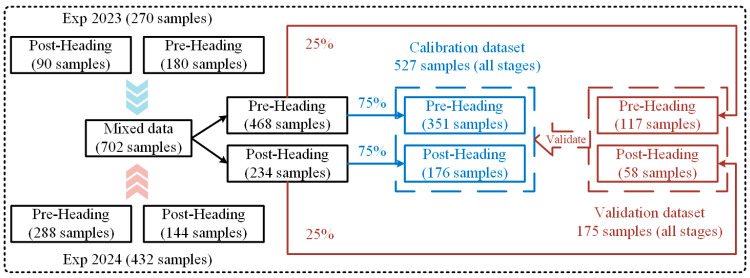
Flowchart of the division of the rice AGB training and test set (from left to right).

This study combined the rice AGB experimental data from 2023 and 2024 to create a comprehensive dataset with high data variability. To analyze the distinct features of various rice growth phases, the combined data were split into two subsets: pre-heading (including tillering, jointing, and booting) and post-heading (including heading). From each of these subsets, 75% of the data was randomly chosen to serve as the training set, and these training sets were then combined to create a comprehensive training set for all growth stages. The remaining 25% from each subset was allocated as the test set for validating the model. The process of data division is depicted in Figure 2.

The statistical results of the training and test sets categorized by different growth stages are shown in Table 2. During the pre-heading stage, AGB exhibited greater variability with a coefficient of variation (CV) of 37.6% for the training set and 36.7% for the test set. In contrast, the post-heading stage demonstrated relatively lower variability, with CV values of 25.9% for the training set and 24.2% for the test set. Notably, both the training and test sets maintained a high degree of consistency in their overall distributions. This consistency not only validates the rationality of the data division but also highlights the significant impact of growth stages on rice canopy structure. Overall, the rice AGB experiment provides a suitable dataset, characterized by extensive variability, which is instrumental in enhancing and evaluating the robustness of subsequent models.

### 2.3. UAV Image Acquisition and Processing

**Figure 3:**
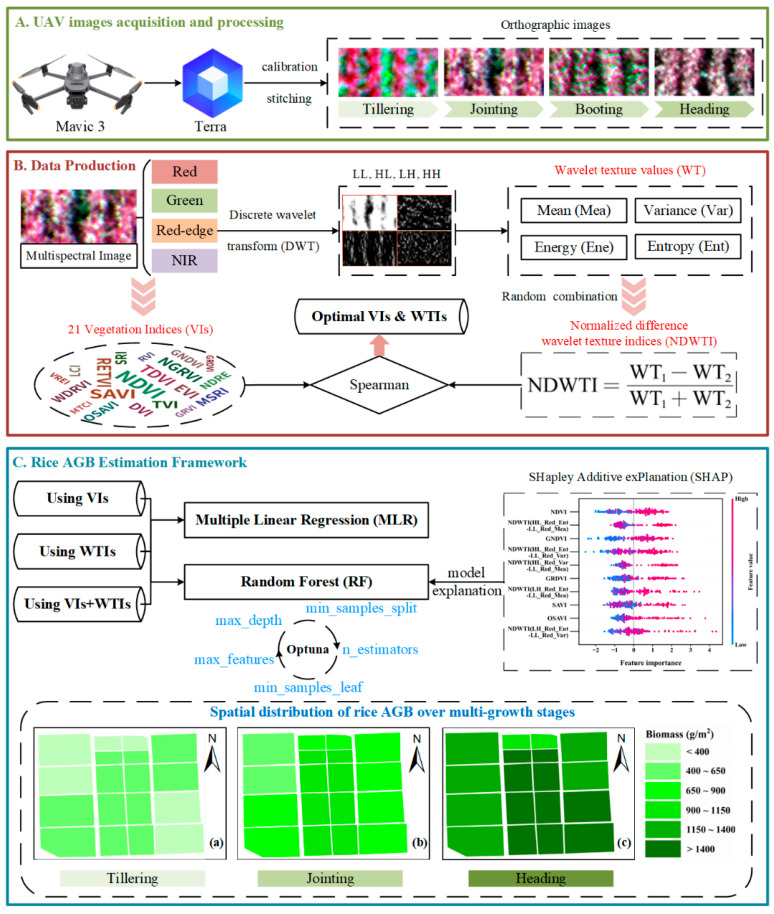
Flowchart of this study. (**A**) Use the UAV equipped with a multispectral sensor to obtain image data of rice tillering, jointing, boosting, and heading stages. (**B**) Calculate 21 VIs from multispectral images, extract WTs, and construct WTIs. (**C**) Framework for constructing the rice AGB estimation model.

Over a two-year experimental timeframe, this research employed the DJI Mavic 3M to capture multispectral imagery of rice canopies at various growth stages (Table 1 and Figure 3A). The DJI Mavic 3M features a visible light camera alongside four 1/2.8-inch CMOS sensors (green, red, red-edge, and near-infrared), with each multispectral sensor boasting 5 million effective pixels. To maintain high-quality image data, all flights were scheduled between 10:00 and 12:00, ensuring clear weather, low wind speeds, and consistent lighting. The UAV operated at a constant altitude of 30 m, with heading and lateral overlap rates set at 80% to meet the precision requirements for post-processing image stitching. Following the data collection, the DJI Terra V5.0.2 software was utilized to preprocess the multispectral information. This software automatically extracts positioning and attitude system data, along with camera settings from the imported multispectral images, and integrates them with standard whiteboard images taken before each flight for radiometric calibration, image stitching, and reflectance calculations, resulting in the creation of multispectral orthoimages. Afterward, ENVI 5.3 software was employed to identify regions of interest (ROIs) within the sampling area, allowing for the extraction of average reflectance values from all pixels in each ROI, which served as the spectral value for that area. Utilizing the acquired multispectral data, the study computed a total of 21 VIs (Figure 3B), with their respective formulas detailed in Supplementary Materials Table S1.

### 2.4. Texture Analysis

#### 2.4.1. Texture Extraction Based on Discrete Wavelet Transform (DWT)

The discrete wavelet transform (DWT) serves as a binary multiscale decomposition technique that is extensively utilized in areas such as signal processing, data compression, and feature extraction [37]. Since the energy of wavelet functions $\Psi \left(t\right)$ typically decays rapidly over time, a series of functions can be generated through scaling and translation using the scale parameter u and shift parameter v [39], as shown in Equation (2):

(2) $$\Psi_{u,v}\left(t\right)=\frac{1}{\sqrt{u}}\Psi \left(\frac{t-v}{u}\right)\left(u,v\in R,u\neq 0\right)$$

where u is the scale parameter and v is the shift parameter.

DWT discretizes u and v based on the above Formula (2), where $u=u_{0}^{m}\left(u_{0}>1\right)$, $v=nv_{0}u_{0}^{m}\left(v_{0}\in R,\left(m,n\right)\in Z^{2}\right)$. In general, $u_{0}=2$ and $v_{0}=1$. If $f\left(t\right)\in L^{2}\left(R\right)$ is a finite energy signal, the DWT function can be expressed as follows:

(3) $$D_{f}\left(m,n\right)=\int_{-\infty }^{\infty }f\left(t\right)\bar{\Psi_{m,n}\left(t\right)}dt$$

where $\bar{\Psi_{m,n}\left(t\right)}$ is the complex conjugate function of $\Psi_{m,n}\left(t\right)$.

In the context of two-dimensional image analysis, the DWT systematically breaks down the image into various frequency subbands through a series of high-pass (H) and low-pass (L) filters, effectively capturing both the outlines and intricate texture details. Each level of decomposition yields three high-frequency subimages: horizontal (LH), vertical (HL), and diagonal (HH), along with one low-frequency subimage (LL) [40]. The choice of wavelet basis functions significantly influences the DWT’s performance and applicability. Among various options, the first-order Haar wavelet transform stands out for its simplicity and efficiency in handling two-dimensional images, offering faster processing speeds [18]. Haar can be defined as follows:

(4) $$\Psi \left(t\right)=\left\{\begin{array}{l} 1;\;0\leq t\leq \frac{1}{2}\\ -1;\;\frac{1}{2}\leq t<1\\ 0;\;otherwise \end{array}\right.$$

**Figure 4:**
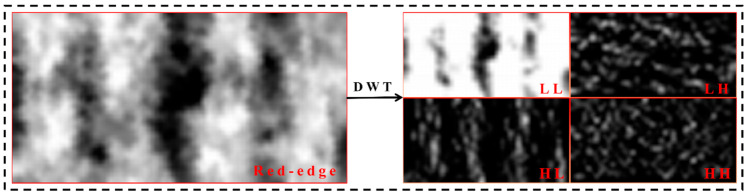
Process of discrete wavelet decomposition of a two-dimensional image (using a multispectral red-edge image as an example).

Consequently, this research utilized the first-order Haar wavelet transform to analyze rice multispectral images across different bands, producing four sub-images: LL, HL, LH, and HH. The decomposition procedure is illustrated in Figure 4, using the red-edge band image as a reference. To enhance feature extraction from the images, standard statistical measures such as mean (Mea), variance (Var), energy (Ene), and entropy (Ent) are computed from the four sub-images (Figure 3B), with the relevant formulas detailed in Equations (5)–(8). To streamline the representation of subsequent wavelet texture features (WTs), various bands, sub-images, and texture descriptors are amalgamated. For instance, LL_Red_Mea denotes the mean texture of the low-frequency sub-image corresponding to the red band. The equations are as follows:

(5) $$Mea=\frac{1}{MN}\sum_{i=1}^{M}\sum_{j=1}^{N}\left(I_{\left(i,j\right)}^{\Upsilon }\right)$$

(6) $$Var=\frac{1}{MN}\sum_{i=1}^{M}\sum_{j=1}^{N}{\left(I_{\left(i,j\right)}^{\Upsilon }-Mea\right)}^{2}$$

(7) $$Ene=\frac{1}{MN}\sum_{i=1}^{M}\sum_{j=1}^{N}{\left(I_{\left(i,j\right)}^{\Upsilon }\right)}^{2}$$

(8) $$Ent=-\frac{1}{MN}\sum_{i=1}^{M}\sum_{j=1}^{N}I_{\left(i,j\right)}^{\Upsilon }\left(\log_{2}\left(I_{\left(i,j\right)}^{\Upsilon }\right)\right)$$

where I represents the image after DWT processing, M and N represents the sizes of the image, $\left(i,j\right)$ represents the image pixel points, $\Upsilon =\left[LL,LH,HL,HH\right]$.

#### 2.4.2. Definition of Normalized Difference Wavelet Texture Index (NDWTI)

In this study, we defined a novel wavelet texture index termed the normalized difference wavelet texture index (NDWTI). Its design aligns with the definition of the traditional normalized difference vegetation index (NDVI), effectively reducing interference from variations in light, background, and overall brightness [41,42]. This index is based on the WTs of four multispectral band images, constructing all possible pairwise combinations to explore their ability to estimate AGB at different growth stages of rice. The complexity of spatial texture is characterized by quantifying the relative differences between high-frequency details and low-frequency structures in rice canopy images, to compensate for the inadequacies of spectral information in spatial morphology characterization. A total of 4032 combinations were obtained for each stage, including 2256 combinations of high-frequency with high-frequency textures, 240 combinations of low-frequency with low-frequency textures, and 1536 combinations of low-frequency with high-frequency textures. The three growth stages (pre-heading, post-heading, and all-stage) in this study resulted in a total of 12,096 pairs of combinations. The definition of NDWTI is as follows:

(9) $$NDWTI=\frac{WT_{1}-WT_{2}}{WT_{1}+WT_{2}}$$

where $WT_{1}$ and $WT_{2}$ are different wavelet texture features.

However, we also observed a significant scale disparity between the WTs extracted from high-frequency subgraphs and those from low-frequency subgraphs, as shown in Section 2.4.1. Taking the variance and entropy of red-band images as examples (Supplementary Materials Figure S1), we found that the numerical values of low-frequency wavelet textures are higher, whereas those of high-frequency wavelet textures are lower. Additionally, there are also notable differences in magnitude among various texture features within the same band. This inconsistency in numerical scale between features is highly likely to result in the contributions of texture features with smaller values being overshadowed by those with larger values when constructing the WTIs. Therefore, in this study, before constructing the NDWTI, we applied the MinMaxScaler module to perform maximum-minimum value normalization on all WTs, effectively unifying the numerical ranges of all texture features.

### 2.5. Methods for Analyzing the Correlation Between Remote Sensing Variables and Rice AGB

The reliability of input variables has a significant impact on the accuracy of rice AGB inversion models. To identify variables that are strongly correlated with AGB and enhance model inversion accuracy, this study used Spearman’s correlation coefficient to assess the correlation between remote sensing variables and AGB. Compared to the Pearson correlation coefficient method, the Spearman correlation coefficient method is independent of data distribution and exhibits good sensitivity to monotonic relationships (both linear and nonlinear), as well as strong robustness to outliers [43]. Additionally, this study employed the Shapiro–Wilk test [29] to assess the normality of remote sensing variables and AGB (Supplementary Materials Figure S2), revealing that most remote sensing variables and AGB data significantly deviated from normal distribution across different growth stages (p<0.05). Given this non-normal distribution, it can be further argued that the choice of the Spearman correlation analysis is consistent with the statistical characteristics of the data in this study, making it more effective for exploring the relationship between remote sensing variables and AGB.

### 2.6. Construction and Evaluation of Rice AGB Prediction Models

This study used multiple linear regression (MLR) and random forest regression (RFR) to train the model (Figure 3C), where the formula for MLR is shown in Equation (10). Equation (10) is as follows:

(10) $$y=\sum_{i=1}^{n}a_{i}x_{i}+a_{0}$$

where y represents the value of the target variable (e.g., AGB), n represents the total number of input variables, i represents the current number of input variables, $x_{i}$ represents the value of the feature variable (e.g., NDVI), $a_{i}$ and $a_{0}$ are the regression coefficient and bias term of the i-th feature variable, respectively, which can be fitted and determined during model training.

RFR is a powerful ensemble machine learning technique that performs regression analysis by creating several decision trees and combining their predictions [44]. Each tree is trained separately on a randomly selected portion of the dataset during the training phase. The overall prediction is derived by averaging or assigning weights to the outputs of the individual trees, which helps to minimize the likelihood of overfitting. The effectiveness of this algorithm is significantly influenced by hyperparameters like n_estimators, max_features, max_depth, min_samples_split, and min_samples_leaf. While intricate models may overfit due to their numerous parameters, simpler models might fail to adequately capture the nonlinear characteristics of the data.

To optimize hyperparameters, this research utilizes the Optuna open-source framework, which employs the sequential model-based optimization (SMBO) technique along with the tree-structured Parzen estimator (TPE) intelligent search strategy to efficiently explore and identify optimal parameter combinations within a specified search space [45]. In this approach, the objective function takes hyperparameters as inputs and returns a target value to optimize the given search space, enabling automated, highly scalable, and computationally efficient hyperparameter search [46]. The hyperparameters considered in this study include the n_estimators, max_features, max_depth, min_samples_split, and min_samples_leaf. The search ranges for these hyperparameters are shown in Supplementary Materials Table S2. Furthermore, the search process incorporates 10-fold cross-validation [47] to ensure a thorough exploration of parameters while improving the model’s generalization through repeated sampling validation. This strategy effectively overcomes the drawbacks of conventional grid and random search methods, which often suffer from high computational demands, inefficiency, and a tendency to settle on local optima. Additionally, all input features are standardized before training to lessen the effects of dimensional differences on the model’s performance.

Use the coefficient of determination ($R^{2}$) and root mean square error (RMSE) to evaluate model performance. Higher $R^{2}$ values and lower RMSE values indicate greater accuracy in model estimation. The calculations for $R^{2}$ and RMSE are as follows:

(11) $$R^{2}=1-\frac{\sum_{i=1}^{n}{\left({\hat{y}}_{i}-y_{i}\right)}^{2}}{\sum_{i=1}^{n}{\left(y_{i}-\bar{y}\right)}^{2}}$$

(12) $$RMSE=\sqrt{\frac{\sum_{i=1}^{n}{\left({\hat{y}}_{i}-y_{i}\right)}^{2}}{n}}$$

where $y_{i}$ and ${\hat{y}}_{i}$ are the measured and estimated values of rice AGB, respectively, $\bar{y}$ is the average value of rice AGB, and n is the total number of samples.

### 2.7. Model Visualization and Explanation Based on Shapley Additive Explanations (SHAP)

Explainable machine learning seeks to address the interpretability challenges posed by the ‘black box’ nature of traditional machine learning. It elucidates how models produce predictive results by uncovering the intrinsic relationships between input features and output outcomes, while also identifying the feature factors that most significantly influence these predictive outcomes [48]. To reflect the impact of changes in input features on the results in the AGB prediction model for different growth stages of rice, this study introduced the SHAP method to perform an explainability analysis of the RFR model (Figure 3C). SHAP is a model explanation framework based on the Shapley concept from game theory [49].

The SHAP values for the input features are computed to measure the individual impact of each feature on predicting rice AGB. To improve the comparability of these contributions, this research utilizes the Z-score technique to transform all SHAP numerical values into standardized indicators that align with the standard normal distribution, thus removing unit discrepancies. The calculation formula is as follows:

(13) $$Z=\frac{x_{i}-\mu }{\sigma }$$

where $x_{i}$ is the *i*-th variable, μ and σ are the mean and standard deviation, respectively.

## 3. Results

### 3.1. Analysis of the Relationship Between VIs and AGB at Different Growth Stages of Rice

**Figure 5:**
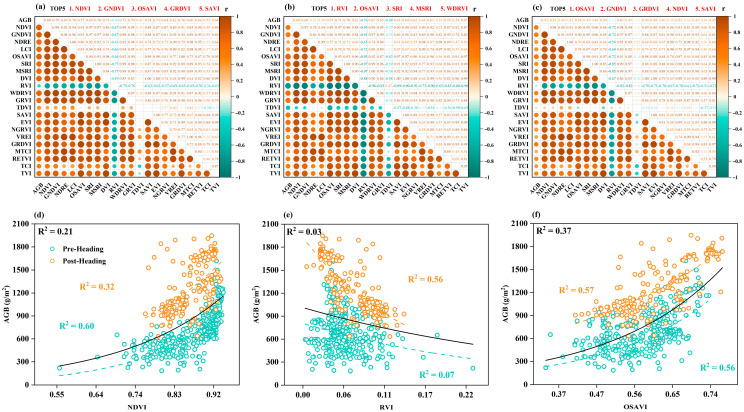
Correlation analysis results and relationship diagrams between VIs and rice AGB. (**a**–**c**) show the correlation between VIs and rice AGB at pre-heading, post-heading, and all-stage, respectively. (**d**–**f**) show the relationship diagrams between the VIs with the highest correlation and rice AGB at pre-heading, post-heading, and all-stage, respectively.

Utilizing the training dataset, we computed the Spearman correlation coefficients for 21 VIs with AGB across various growth stages to evaluate the effectiveness of VIs in estimating AGB. The findings from the correlation analysis for the pre-heading, post-heading, and all-stage are illustrated in Figure 5a–c. A majority of the VIs showed a strong correlation with AGB at different growth stages. However, significant variations in correlation were observed depending on the growth stage. In particular, VIs exhibited robust correlations with AGB during the pre-heading stage, whereas these correlations tended to diminish in the post-heading stage. This stage-dependent fluctuation contributed to an overall decrease in the correlation between VIs and AGB across all growth stages, suggesting that the responsiveness of VIs to AGB lessened as the growth stage advanced. The VIs that displayed the strongest correlations at each growth phase were NDVI (|r| = 0.80), RVI (|r| = 0.76), and OSAVI (|r| = 0.58). To further assess the ability of VIs to estimate AGB, we developed simple regression models for AGB estimation based on the VIs with the highest correlations at each stage, as shown in Figure 5d–f. Overall, the accuracy of AGB estimation models created using VIs across all growth stages was notably lower than those tailored for the pre-heading or post-heading stages. While OSAVI showed relatively consistent estimation performance across all growth stages (R^2^ = 0.37), its accuracy was significantly inferior to that of stage-specific models (e.g., pre-heading: R^2^ = 0.56, post-heading: R^2^ = 0.57), and it was not the optimal feature for estimating AGB in a single growth stage. This aligns with the above correlation analysis results and highlights that the dynamic alterations in canopy structure and spectral responses at various growth stages make it challenging to utilize a single VI for biomass estimation across all stages of growth. Ultimately, we identified the five VIs with the strongest correlations to AGB at various growth stages to achieve a balance between stage specificity and feature diversity, which will serve as input variables for the subsequent AGB inversion models.

### 3.2. Analysis of the Relationship Between WTs and AGB at Different Stages of Rice Growth

**Figure 6:**
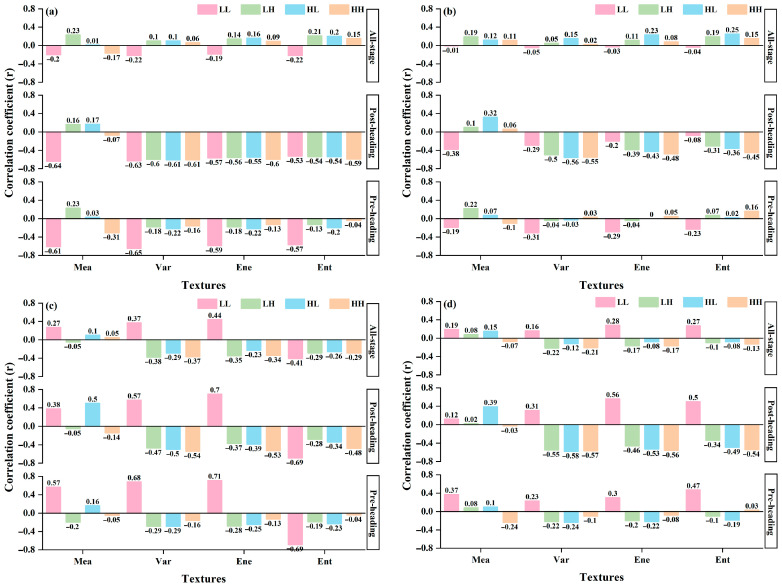
Analysis of the correlation between WTs and AGB across various growth stages of rice. (**a**–**d**) show the WTs of red, green, near-infrared and nir images, respectively.

The correlation analysis results, derived from the training dataset, illustrate the relationship between the texture features obtained from multispectral images and AGB at various growth stages, as depicted in Figure 6. At pre-heading, the low-frequency texture features extracted from images of different bands generally demonstrated higher correlations with AGB than high-frequency texture features. Among these, low-frequency texture features from the near-infrared band exhibited higher correlations, with the highest correlation achieved by LL_NIR_Ene (|r| = 0.71). Following this, the red band presents a significant correlation achieved by LL_Red_Var (|r| = 0.65). In contrast, high-frequency texture features maintained correlations below 0.4, with the highest correlation being HH_Red_Mea (|r| = 0.31). It is important to highlight that the correlation between high-frequency texture features and AGB increased at post-heading and all-stage, with some high-frequency texture features even outperforming low-frequency texture features. Specifically, at post-heading, the correlations for high-frequency features were notably higher than those observed at pre-heading. The strongest correlations at post-heading were LL_NIR_Ene (|r| = 0.70) for low-frequency and HH_Red_Var (|r| = 0.61) for high-frequency features. For the all-stage analysis, the top correlations were LL_NIR_Ene (|r| = 0.44) and HH_NIR_Var (|r| = 0.37), respectively. Overall, both high-frequency and low-frequency texture features exhibited relatively weak correlations with AGB across pre-heading, post-heading, and all-stage. The high correlation of low-frequency texture features with AGB at the pre-heading suggests that low-frequency texture features may be more effective for estimating AGB during the vegetative growth stage of rice. Conversely, the increased correlation of high-frequency texture features with AGB at the post-heading and all-stage indicated that high-frequency texture features have certain application potential for estimating AGB during the reproductive growth stage of rice.

**Figure 7:**
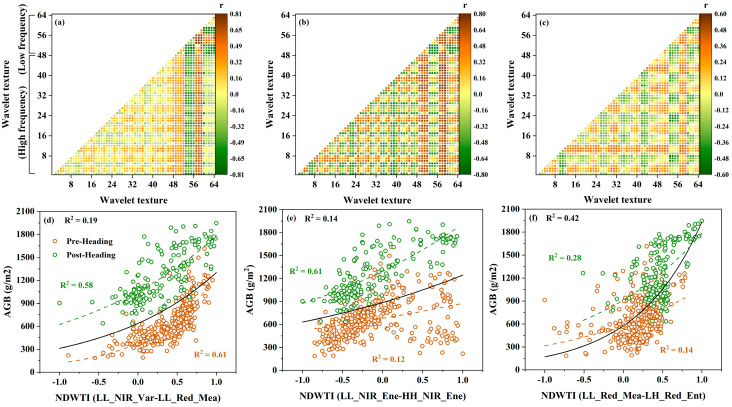
Correlation analysis results and relationship diagrams between NDWTIs and rice AGB. (**a**–**c**) show the correlation between NDWTIs and rice AGB at pre-heading, post-heading, and all-stage, respectively. (**d**–**f**) show the relationship diagrams between the NDWTI with the highest correlation and rice AGB at pre-heading, post-heading, and all-stage, respectively.

Given the weak relationship observed among different WTs and AGB across various growth stages, this study developed a new index, NDWTI, by integrating multiple WTs to enhance texture analysis for estimating rice AGB. The correlation analysis results for all NDWTIs and AGB at pre-heading, post-heading, and all-stage are illustrated in Figure 7a–c, with the ten combinations showing the strongest correlations detailed in Table 3. During the pre-heading stage, combinations of low-frequency texture features exhibited notable effectiveness, particularly those in the near-infrared and red bands, with NDWTI (LL_NIR_Var-LL_Red_Mea) achieving the highest correlation (|r| = 0.81). In the post-heading and all-stage, NDWTI combinations that correlated well with AGB included both low-frequency and high-frequency texture features. Specifically, for the post-heading stage, the leading combinations were those that integrated low and high-frequency features in the near-infrared band, with NDWTI (LL_NIR_Ene-HH_NIR_Ene) showing the strongest correlation (|r| = 0.79). For all-stage, the best-performing combinations were those that combined low and high-frequency features in the red band, with NDWTI (LL_Red_Mea-LH_Red_Ent) achieving the highest correlation (|r| = 0.60). Conversely, NDWTI formed from high-frequency feature combinations yielded lower correlations with AGB throughout all growth stages. Nonetheless, high-frequency texture features showed considerable potential when used alongside low-frequency features during the later growth stages and all stages. In summary, NDWTI significantly enhanced the correlation with AGB across various growth stages compared to individual texture features, with its best combination surpassing the correlation coefficients of VIs. Furthermore, the simple regression models for AGB estimation based on the optimal NDWTI across different growth stages are depicted in Figure 7d–f. NDWTI provided superior AGB estimation accuracy across all growth stages (R^2^ = 0.61, R^2^ = 0.61, and R^2^ = 0.42), outperforming VIs (R^2^ = 0.60, R^2^ = 0.56, and R^2^ = 0.37). These findings suggest that constructing NDWTI from WTs across various frequency domains using multispectral images can effectively capture changes in the spatial structure and details of the rice canopy, leading to more precise AGB estimations. Notably, during the later growth stages when VIs show reduced sensitivity to AGB, NDWTI continues to provide reliable estimation performance. Similarly to VIs, the top five NDWTIs with the highest correlation coefficients at different growth stages were chosen as inputs for subsequent models.

### 3.3. Construction and Validation of the Model for Estimating AGB in Rice

The effectiveness of basic regression models utilizing optimal VI and NDWTI for estimating AGB was assessed with a separate test set across three distinct growth phases of rice (Table 4). During the pre-heading stage, VI demonstrated the best validation performance. Compared to VI, NDWTI exhibited better estimation capabilities during the post-heading stage and all-stage. However, the validation performance of both single features was unsatisfactory at all-stage. In summary, simple regression models based on single features exhibit significant limitations in estimating rice AGB, particularly when integrating data across all growth stages, where their explanatory power is markedly diminished.

**Figure 8:**
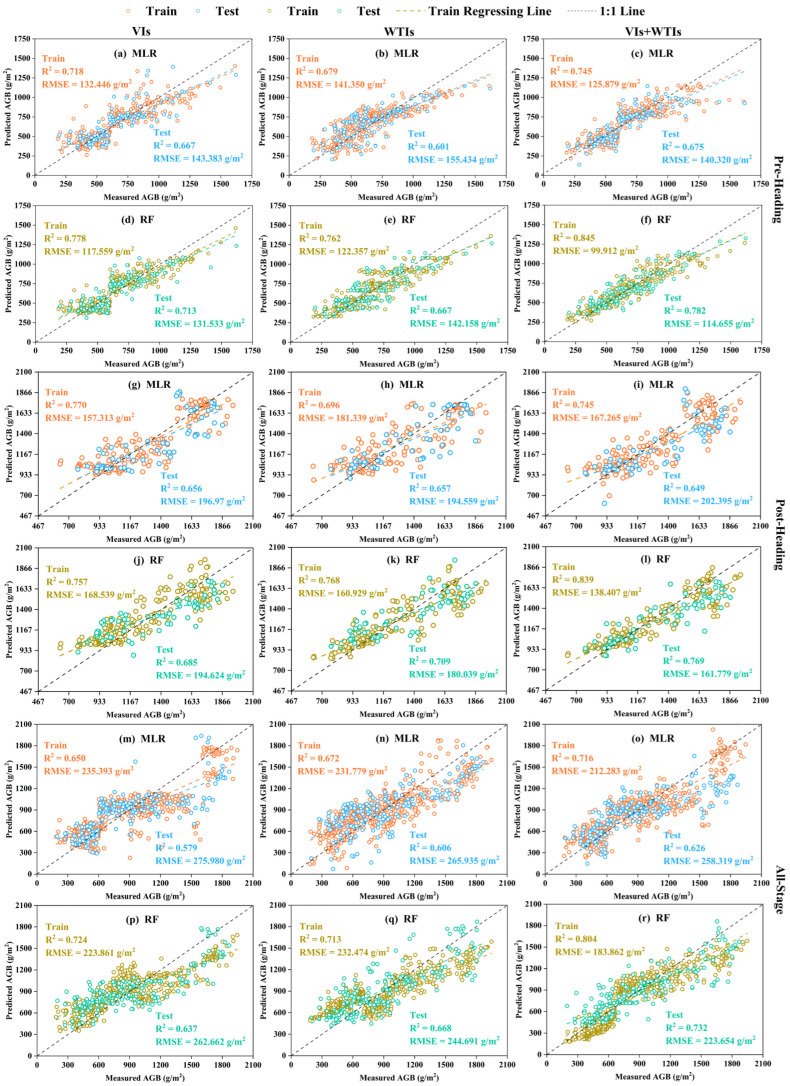
Rice AGB estimation model based on MLR and RF algorithms. (**a**–**c**,**g**–**i**,**m**–**o**) show the model results of MLR combined with three feature datasets at three different growth stages, respectively. (**d**–**f**,**j**–**l**,**p**–**r**) show the model results of RF combined with three feature datasets at three different growth stages, respectively. The training and testing datasets are distinguished by different colored scatter points.

The top five VIs and NDWTIs selected at different growth stages in the above analysis were divided into three feature datasets (VIs, NDWTIs, VIs+NDWTIs) as input variables. MLR and Optuna-RF algorithms were used to construct AGB estimation models for different growth stages of rice (Figure 8). The findings reveal that the AGB estimation models created using either MLR or Optuna-RF, based on VIs or NDWTIs, significantly outperformed the simple regression model across all growth stages. Notably, the Optuna-RF model achieved superior performance metrics, exhibiting a higher R^2^ and a lower RMSE compared to the MLR model. Scatter plots illustrating the relationship between predicted and actual values (e.g., Figure 8h,k) indicated that the predictions from the Optuna-RF model were more tightly clustered around the 1:1 line, whereas those from the MLR model showed greater dispersion. To further improve AGB estimation across different growth stages, models were developed by combining VIs and NDWTIs. The results from both MLR and Optuna-RF demonstrated that multi-feature fusion models outperformed single-feature models at various growth stages (e.g., Figure 8c,f). For instance, the Optuna-RF model utilizing both VIs and NDWTIs enhanced the validation R^2^ for AGB estimation by 14.91% and 9.58% compared to using VIs and NDWTIs alone, respectively, while also decreasing the validation RMSE by 14.85% and 9.41%. In contrast, the MLR model showed only marginal improvements, with some instances of reduced accuracy (Figure 8i). Throughout the three growth stages, Optuna-RF (R^2^ = 0.732–0.782) consistently surpassed MLR in performance (R^2^ = 0.626–0.714). These outcomes not only confirm the utility of NDWTI for AGB estimation at various rice growth stages but also highlight the significant impact of integrating multiple features in AGB estimation.

**Figure 9:**
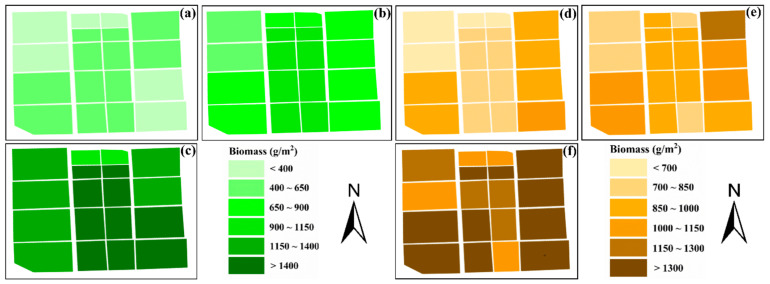
AGB (g/m^2^) map of rice based on Optuna-RF combined with VIs+NDWTIs. (**a**–**c**) show the distribution maps of AGB at pre-heading, post-heading, and all-stage of rice in 2023, respectively, while (**d**–**f**) show the distribution maps of AGB at pre-heading, post-heading, and all-stage of rice in 2024, respectively.

To assess the real-world utility of the model and analyze the variations in the spatial distribution of rice AGB in the field, a combination of Optuna-RF and various data sources was employed to create maps of AGB across different growth stages of rice over a two-year experimental period. Figure 9 illustrates that AGB shows a steady increase as the rice growth stage progresses, aligning closely with findings from field surveys. This effectively showcases the effectiveness of UAV multispectral remote sensing technology for tracking rice crop development in agricultural settings.

### 3.4. Contribution of Spectral and Textural Features to AGB Estimation for Rice

**Figure 10:**
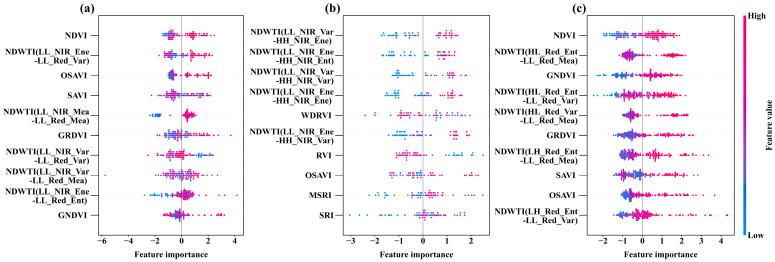
SHAP summary plot of the rice AGB estimation model based on Optuna-RF. Each point represents a feature value and its corresponding SHAP value. Zero SHAP values are used as the boundary line; samples to the left harm the estimated value, while those to the right have a positive impact. Colors indicate the magnitude of the feature values. (**a**) Pre-heading, (**b**) post-heading, (**c**) all-stage.

To enhance the understanding of how various features influence the estimation of rice AGB across different growth phases, an interpretability analysis was conducted using the SHAP method on the feature variables integrated into Optuna-RF. This analysis clarified the connection between feature values and their respective SHAP values (Figure 10). Findings reveal that VIs play a crucial role in AGB estimation before the heading stage, where elevated VI values positively affect the model’s output (Figure 10a), with NDVI being the most influential. Conversely, after heading, the significance of WTIs markedly increased, with higher values correlating with a more substantial positive effect on the model, particularly highlighted by NDWTI (LL_NIR_Var-HH_NIR_Ene) (Figure 10b). For all-stage, the synergistic effect of VIs and WTIs makes a significant contribution to AGB estimation, demonstrating that increases in both variables lead to a greater positive impact on model output (Figure 10c). These results further validate the advantages of texture analysis, particularly the NDWTI proposed in this study, for estimating AGB at post-heading and all-stage of rice, underscoring the necessity of dynamically selecting key features based on different growth stages.

## 4. Discussion

### 4.1. Limitations of VIs in Estimating AGB

Spectral information derived from multispectral images obtained by UAV has been extensively utilized in research focused on crop biomass and other growth monitoring [50]. As a key derivative parameter of spectral information, VIs combine information from different wavelengths to effectively enhance vegetation feature signals while suppressing background noise. These indices have emerged as vital tools for quantitative remote sensing in agricultural studies [51,52]. In this research, the chosen VIs exhibited strong correlations with AGB at various rice growth stages. Notably, the model’s accuracy peaked during the pre-heading stage but showed a decline in the post-heading and all-stage. This observation is consistent with Zhang’s findings [21], which noted a reduction in VI effectiveness for tracking crop growth metrics in later growth phases. This decline may be attributed to the fact that before the heading stage, rice is in the vegetative growth phase, characterized by rapid increases in leaf area that have not yet reached saturation. At this stage, the canopy structure is relatively simple, with leaves distributed uniformly, and leaves exert the most significant influence on canopy spectral characteristics. This enables VIs such as the NDVI, which are sensitive to leaf area, to exhibit strong explanatory power. In the later growth stage, rice transitions from vegetative growth to reproductive growth, not only because leaf area increases toward saturation, reducing spectral variability sensitivity, but also due to the biomass allocation patterns shifting from leaves to panicles. The complex canopy structure, composed of stems, leaves, and panicles, further affects the monitoring performance of VIs. Although previous studies have indicated that the accuracy of rice AGB estimation after heading can be improved using hyperspectral data [53,54]. However, the limited number of bands in multispectral cameras restricts the selection of available VIs. The current results suggest that estimating rice AGB using solely multispectral VIs, especially after heading and all stages, is constrained. Therefore, extracting additional feature information from UAV multispectral images is of great significance for improving the accuracy of rice AGB estimation.

### 4.2. Advantages of DWT

Texture analysis addresses some of the limitations posed by the limited number of multispectral bands by quantifying spatial characteristics, which enhances the dimensionality of information and the ability to express features within the data [55]. This method proves to be a valuable technical tool for precision agriculture that employs multispectral images obtained from UAVs. In our research, we opted for DWT to derive texture features instead of the commonly used GLCM-based method. This choice stems from the fact that GLCM’s effectiveness is heavily reliant on predetermined window sizes and analysis directions, leading to reduced flexibility and increased computational demands. However, wavelet textures preserve overall structural information while providing enhanced capabilities for multi-directional detail acquisition and superior computational efficiency. We think that wavelet texture analysis could provide an alternative, feasible approach for quantifying crop spatial structural features. The analysis of WTs calculated at different growth stages and AGB in this study revealed that, except for the red and near-infrared bands, most wavelet texture features exhibited weak correlations with AGB, consistent with previous conclusions using texture features to estimate biomass [21,56]. Additionally, we found that before heading, low-frequency texture features exhibited higher correlations with AGB compared to high-frequency texture features. However, after heading, high-frequency texture features demonstrated significantly higher correlations with AGB than before heading. This may be attributed to the relatively uniform structure of the rice canopy before heading, where leaves are arranged in an orderly manner and overlap extensively. Therefore, low-frequency textures can more effectively capture the overall distribution and structural information of the rice canopy, thereby exhibiting a strong correlation with AGB. After heading, the emergence of rice panicles complicates the canopy structure, leading to increased local grayscale variations and texture details in the image. Related studies have also indicated that high-frequency information can effectively reflect changes in image detail features and have been applied to crop growth monitoring in potatoes [38] and rice [29]. Given that individual wavelet textures were inadequate for estimating AGB across different growth stages of rice, we introduced the NDWTI, which integrates various wavelet textures to enhance their performance in estimating rice AGB. The findings revealed that NDWTIs significantly improved the correlation and accuracy of AGB estimations at various growth stages compared to single texture features. The optimal NDWTI for post-heading and all-stage collectively outperformed all evaluated VIs, demonstrating that NDWTI effectively reduces the impacts of soil background, sun angle, and sensor angle by normalizing different texture features, thus enhancing their responsiveness to changes in AGB. These findings are in agreement with the conclusions drawn by Zheng [56]. However, the difference lies in the fact that we further investigated the dynamic response characteristics of low-frequency and high-frequency components of rice canopy texture at different growth stages and found that the combination of low-frequency and high-frequency texture features is complementary at different growth stages of rice, especially for post-heading and all-stage. This further illustrates that the NDWTI proposed in this study not only balances structural and detailed information but also comprehensively reflects changes in canopy morphology throughout the growth process, providing a new approach to construct texture features for quantitative monitoring of biomass and other parameters in agricultural remote sensing through texture analysis.

### 4.3. Complementary Analysis of VIs and NDWTIs

VIs and texture are two important features in remote sensing, each possessing distinct advantages and complementary values in the quantitative monitoring of crop growth. VIs primarily reflect biochemical parameters such as the content of photosynthetic pigments in leaves, whereas texture features are more effective in capturing the spatial structural information of the canopy [57]. This fundamental difference provides a theoretical foundation for their synergistic effects, which are particularly evident during different growth stages of rice. In this study, models based on VIs performed better at pre-heading, while models based on NDWTIs demonstrated higher estimation accuracy for post-heading and all-stage. However, it is important to highlight that employing only single-type features, such as VIs or NDWTIs, did not result in the highest accuracy for AGB estimation models at various growth stages. Conversely, the integration of spectral and texture features significantly enhanced the accuracy of AGB estimations during the different growth stages of rice. Notably, the RF model that combined both spectral and texture features delivered the most optimal estimation results (Figure 8f,l,r). These results further confirm that the combination of spectral and textural features can significantly enhance the performance of AGB estimation, consistent with previous findings in AGB estimation studies on corn [58], potato [7], wheat [59], and rice [60]. Additionally, we think that texture features can compensate for the limitations of single spectral features during the reproductive growth stage of rice. Especially after the heading stage, when VIs tend to saturate, the spatial texture information provided by NDWTIs becomes a key factor in improving model performance (Figure 8p,r). SHAP analysis further validated that VIs significantly contribute to estimating rice AGB before the heading stage. After the heading stage, NDWTIs emerge as vital variables influencing the accuracy of AGB estimation. However, they cannot entirely supplant the synergistic effects of both feature types, as evidenced by the SHAP analysis conducted throughout the all-growth stage.

### 4.4. Comparison of MLR and RF Performance in Estimating AGB

MLR, a traditional approach in linear modeling, is often utilized in research focused on the estimation of crop growth parameters due to its quick modeling capabilities, straightforwardness, and interpretability [37,61]. In this study, MLR achieved acceptable accuracy in estimating AGB at different growth stages when using datasets based on VIs or NDWTIs, significantly enhancing precision compared to simple regression models established with a single VI (e.g., NDVI) or NDWTI (e.g., NDWTI (LL_NIR_Var-LL_Red_Mea)). However, the results obtained from MLR combined with VIs and NDWTIs at different growth stages were unsatisfactory, with little improvement in model accuracy and even a decline in accuracy in some cases (Figure 8i). On one hand, we think that the large number of input variables in the MLR model may increase model complexity, leading to overfitting. On the other hand, since MLR is a linear assumption model, it struggles to effectively capture the nonlinear relationships between numerous features and AGB [62].

In contrast, RF utilizes an ensemble approach by creating several distinct decision trees, which can autonomously detect nonlinear relationships among variables. This capability significantly enhances the model’s ability to generalize in high-dimensional feature environments and mitigates the likelihood of overfitting. Additionally, RF exhibits improved robustness when faced with outliers or noisy datasets. Previous research has indicated that RF models are proficient in minimizing noise interference while sustaining consistent predictive accuracy, particularly with extensive datasets that are high-dimensional and nonlinear, often resulting in superior precision [38,63]. In this study, RF achieved higher R^2^ and lower RMSE than MLR when estimating AGB across different growth stages using different datasets (VIs, NDWTIs, and VIs+NDWTIs). This is also attributed to the optimization of the RF model through Optuna, which automatically determines key hyperparameters such as n_estimators, enabling the model to maintain strong fitting capability while avoiding overfitting. Therefore, RF can more effectively identify and handle the complex relationship between remote sensing variables and rice AGB, thereby demonstrating stable predictive performance across different growth stages.

### 4.5. Application Potential and Limitations

This research involved the computation of texture characteristics from multispectral images using DWT, leading to the development of a novel texture index called NDWTI. By combining VIs, effective estimation of AGB at different growth stages of rice was achieved, verifying the feasibility and effectiveness of wavelet texture features for monitoring changes in rice AGB. Compared to complex processes that extract features from hyperspectral or LiDAR data, this method offers higher operational simplicity and lower cost in terms of data acquisition, feature calculation, and model construction, enhancing its applicability in actual agricultural production. Furthermore, this method demonstrates a degree of transferability and can theoretically be extended to monitor the growth of other crops. However, the generalizability of the multispectral feature combination technique used in this study requires further validation. It is necessary to collect and broaden the sample types under different regional, crop variety, and environmental conditions to assess the stability and generalization capability of this method. In the future, we will attempt to apply this approach across various agricultural field trials to investigate the model’s adaptability and its resilience to factors like diverse crops, different varieties, and varying image resolutions. Furthermore, deep learning possesses stronger data mining capabilities compared to machine learning [64]. Subsequent research will also explore feeding raw imagery directly into deep learning models (such as CNN) to automatically extract more latent features, thereby enhancing the ability to characterize the spatial structure and spectral information of the crop canopy.

## 5. Conclusions

This research assessed the precision of estimating rice AGB across different growth stages by integrating VIs and DWT-based texture features extracted from UAV multispectral imagery. Differing from previous studies that mainly focused on spectral indices or single-stage estimation, this study systematically quantified the contributions of VIs and WTs across multiple growth stages, providing a stage-specific understanding of feature importance. The newly introduced wavelet texture index (NDWTI) exhibited exceptional accuracy in estimating AGB, especially during the post-heading and all-stage, surpassing the performance of VIs. Additionally, by refining the random forest (RF) model with the Optuna algorithm and merging VIs with WTs for both training and validation, significant advancements in AGB estimation across various growth stages were realized. The R^2^ values for the validation dataset were recorded at 0.782, 0.769, and 0.732 for pre-heading, post-heading, and all-stage, respectively, with RMSE values of 114.655 g/m^2^, 174.779 g/m^2^, and 223.654 g/m^2^. When compared to the use of VIs alone, the R^2^ values for validation improved by 9.68%, 12.26%, and 14.91% across different growth stages, while RMSE values decreased by 12.83%, 16.88%, and 14.85%, respectively. To further understand the contribution of features to the model’s performance. SHAP analysis quantified the relative importance of VIs and WTs across rice growth stages, indicating that VIs primarily influenced AGB estimation during the vegetative growth stages, whereas WTs became the predominant contributors during the reproductive growth stages. In summary, the NDWTI constructed in this research has proven effective for estimating rice AGB. The integration of WTs with VIs significantly enhances the reliability and generalizability for estimating rice AGB across different growth stages, demonstrating that combining spectral and texture features not only alleviates performance issues related to saturation in later growth stages but also offers a cost-effective and valuable approach for monitoring crop growth dynamics and enhancing precision agricultural practices.

## Figures and Tables

**Table 1:** Experimental design and data collection schedule.

Experiment	Transplanting	Sampling/UAV Test Date	Stage	Altitude
Exp. 2023	28 May.	27 June.	Late-tillering	30 m
		22 July.	Jointing	30 m
		19 August.	Heading	30 m
Exp. 2024	27 May.	19 July.	Jointing	30 m
		3 August.	Booting	30 m
		21 August.	Heading	30 m

**Table 2:** Descriptive statistics of AGB for training and validation datasets at different growth stages of rice.

Stages	AGB (g/m^2^)					
	Number	Min	Max	Average	SD	CV (%)
	Calibration dataset					
Pre-Heading	351	184.560	1613.664	663.026	249.474	37.6
Post-Heading	176	633.120	1947.120	1269.291	329.153	25.9
All-Stage	527	184.560	1947.120	863.578	398.538	46.2
	Validation dataset					
Pre-Heading	117	197.520	1620.240	670.769	246.056	36.7
Post-Heading	58	768.600	1868.160	1354.337	327.127	24.2
All-Stage	175	197.520	1868.160	898.625	424.193	47.2

SD: standard deviation; CV: coefficient of variation.

**Table 3:** Top 10 NDWTIs with high correlation with rice AGB (ranked by |r|).

Pre-Heading			Post-Heading			All-Stages		
WT1	WT2	|r|	WT1	WT2	|r|	WT1	WT2	|r|
LL_NIR_Var	LL_Red_Mea	0.81	LL_NIR_Ene	HH_NIR_Ene	0.79	LL_Red_Mea	LH_Red_Ent	0.60
LL_NIR_Ene	LL_Red_Var	0.80	LL_NIR_Var	HH_NIR_Var	0.78	LL_Red_Mea	HL_Red_Ent	0.58
LL_NIR_Mea	LL_Red_Mea	0.80	LL_NIR_Ene	HH_NIR_Var	0.78	LL_Red_Mea	HL_Red_Var	0.57
LL_NIR_Ene	LL_Red_Ent	0.79	LL_NIR_Var	HH_NIR_Ene	0.78	LL_Red_Var	HL_Red_Ent	0.56
LL_NIR_Var	LL_Red_Var	0.79	LL_NIR_Ene	HH_Red-edge_Ent	0.78	LL_Red_Var	LH_Red_Ent	0.55
LL_Red-edge_Mea	LL_Red_Mea	0.79	LL_NIR_Ene	HH_NIR_Ent	0.77	LL_Red_Mea	HH_Red_Ent	0.50
LL_NIR_Mea	LL_Green_Mea	0.78	LL_NIR_Mea	HH_NIR_Var	0.77	LL_Red_Ent	HL_Red_Ent	0.48
LL_Red-edge_Var	LL_Red_Var	0.78	LL_NIR_Var	HH_NIR_Ent	0.77	LL_Red_Mea	LH_Red_Var	0.48
LL_NIR_Var	LL_Green_Mea	0.78	LL_NIR_Ene	HH_Red-edge_Var	0.77	LL_Red_Var	HL_Red_Var	0.47
LL_NIR_Ene	LL_Green_Var	0.78	LL_NIR_Ene	LH_Red-edge_Var	0.77	LH_NIR_Ene	LH_Red-edge_Ent	0.47

**Table 4:** Validation results of the simple regression model constructed using optimal VI and NDWTI at pre-heading, post-heading, and all-stage.

Stages	AGB(g/m^2^)		
	Variable	R^2^	RMSE
	VIs		
Pre-heading	NDVI	0.544	165.536
Post-heading	RVI	0.509	223.557
All-heading	OSAVI	0.261	367.433
	NDWTIs		
Pre-heading	NDWTI(LL_NIR_Var-LL_Red_Mea)	0.491	174.801
Post-heading	NDWTI(LL_NIR_Ene-HH_NIR_Ene)	0.562	214.580
All-heading	NDWTI(LL_Red_Mea-LH_Red_Ent)	0.359	352.853

## Data Availability

The original contributions presented in this study are included in the article. Further inquiries can be directed to the corresponding author.

## Supplementary Materials

The following supporting information can be downloaded at: https://www.mdpi.com/article/10.3390/plants14182903/s1. Figure S1. Statistical distribution of high-frequency and low-frequency texture features (using variance and entropy of multispectral red band images as examples); Figure S2. Statistical results of normality tests for remote sensing variables at different growth stages of rice and AGB; Table S1. 21 VIs used in this study; Table S2. Summary of hyperparameters for the RF models. References [65,66,67,68,69,70,71,72,73,74,75,76,77,78,79,80,81,82,83] are cited in the Supplementary Materials.

## References

[1] Yuan L.-P. (2014). Development of Hybrid Rice to Ensure Food Security. Rice Sci..

[2] Yang H.W., Zhang L.Y., Gao X.Q., Han S., Ma Z.B., Wang L.L. (2025). Multi-Dimensional Analysis of Quality-Related Traits Affecting the Taste of Main Cultivated Japonica Rice Varieties in Northern China. Agronomy.

[3] Shu M.Y., Zuo J.Y., Shen M.Y., Yin P.F., Wang M., Yang X.H., Tang J.H., Li B.G., Ma Y.T. (2021). Improving the estimation accuracy of SPAD values for maize leaves by removing UAV hyperspectral image backgrounds. Int. J. Remote Sens..

[4] Jin X.L., Madec S., Dutartre D., de Solan B., Comar A., Baret F. (2019). High-Throughput Measurements of Stem Characteristics to Estimate Ear Density and Above-Ground Biomass. Plant Phenomics.

[5] Li Z.H., Zhao Y., Taylor J., Gaulton R., Jin X.L., Song X.Y., Li Z.H., Meng Y., Chen P.F., Feng H.K. (2022). Comparison and transferability of thermal, temporal and phenological-based in-season predictions of above-ground biomass in wheat crops from proximal crop reflectance data. Remote Sens. Environ..

[6] Kumar A., Tewari S., Singh H., Kumar P., Kumar N., Bisht S., Devi S., Nidhi, Kaushal R. (2021). Biomass accumulation and carbon stock in different agroforestry systems prevalent in the Himalayan foothills, India. Curr. Sci..

[7] Liu Y., Feng H.K., Yue J.B., Li Z.H., Yang G.J., Song X.Y., Yang X.D., Zhao Y. (2022). Remote-sensing estimation of potato above-ground biomass based on spectral and spatial features extracted from high-definition digital camera images. Comput. Electron. Agric..

[8] Zhou C.Q., Ye H.B., Hu J., Shi X.Y., Hua S., Yue J.B., Xu Z.F., Yang G.J. (2019). Automated Counting of Rice Panicle by Applying Deep Learning Model to Images from Unmanned Aerial Vehicle Platform. Sensors.

[9] Zhang J.Y., Liu X., Liang Y., Cao Q., Tian Y.C., Zhu Y., Cao W.X., Liu X.J. (2019). Using a Portable Active Sensor to Monitor Growth Parameters and Predict Grain Yield of Winter Wheat. Sensors.

[10] Weiss M., Jacob F., Duveiller G. (2020). Remote sensing for agricultural applications: A meta-review. Remote Sens. Environ..

[11] Meng L., Yin D.M., Cheng M.H., Liu S.B., Bai Y., Liu Y., Liu Y.D., Jia X., Nan F., Song Y. (2023). Improved Crop Biomass Algorithm with Piecewise Function (iCBA-PF) for Maize Using Multi-Source UAV Data. Drones.

[12] Hassan M.A., Yang M.J., Rasheed A., Yang G.J., Reynolds M., Xia X.C., Xiao Y.G., He Z.H. (2019). A rapid monitoring of NDVI across the wheat growth cycle for grain yield prediction using a multi-spectral UAV platform. Plant Sci..

[13] Shu M.Y., Shen M.Y., Dong Q.Z., Yang X.H., Li B.G., Ma Y.T. (2022). Estimating the maize above-ground biomass by constructing the tridimensional concept model based on UAV-based digital and multi-spectral images. Field Crops Res..

[14] Skobalski J., Sagan V., Alifu H., Al Akkad O., Lopes F.A., Grignola F. (2024). Bridging the gap between crop breeding and GeoAI: Soybean yield prediction from multispectral UAV images with transfer learning. Isprs J. Photogramm. Remote Sens..

[15] Derraz R., Muharam F.M., Nurulhuda K., Jaafar N.A., Yap N.K. (2023). Ensemble and single algorithm models to handle multicollinearity of UAV vegetation indices for predicting rice biomass. Comput. Electron. Agric..

[16] Zhao D., Yang H., Yang G.J., Yu F.H., Zhang C.J., Chen R.Q., Tang A.H., Zhang W.J., Yang C., Xu T.Y. (2024). Estimation of Maize Biomass at Multi-Growing Stage Using Stem and Leaf Separation Strategies with 3D Radiative Transfer Model and CNN Transfer Learning. Remote Sens..

[17] Yu F.H., Xiang S., Xu C.Y., Jin Z.Y. (2024). PIOSL-PRO: Construction of the PIOSL Model Considering the Distribution of Multiple Elements. IEEE Trans. Geosci. Remote Sens..

[18] Zhou C., Gong Y., Fang S.H., Yang K.L., Peng Y., Wu X.T., Zhu R.S. (2022). Combining spectral and wavelet texture features for unmanned aerial vehicles remote estimation of rice leaf area index. Front. Plant Sci..

[19] Jin Z.Y., Liu H.Z., Cao H.N., Li S.L., Yu F.H., Xu T.Y. (2025). Hyperspectral Remote Sensing Estimation of Rice Canopy LAI and LCC by UAV Coupled RTM and Machine Learning. Agriculture.

[20] Zheng H.B., Cheng T., Li D., Zhou X., Yao X., Tian Y.C., Cao W.X., Zhu Y. (2018). Evaluation of RGB, Color-Infrared and Multispectral Images Acquired from Unmanned Aerial Systems for the Estimation of Nitrogen Accumulation in Rice. Remote Sens..

[21] Zhang J.Y., Qiu X.L., Wu Y.T., Zhu Y., Cao Q., Liu X.J., Cao W.X. (2021). Combining texture, color, and vegetation indices from fixed-wing UAS imagery to estimate wheat growth parameters using multivariate regression methods. Comput. Electron. Agric..

[22] Yu F.H., Bai J.C., Jin Z.Y., Zhang H.G., Yang J.X., Xu T.Y. (2023). Estimating the rice nitrogen nutrition index based on hyperspectral transform technology. Front. Plant Sci..

[23] Song E.Z., Shao G.C., Zhu X.Y., Zhang W., Dai Y., Lu J. (2024). Estimation of Plant Height and Biomass of Rice Using Unmanned Aerial Vehicle. Agronomy.

[24] Che Y.P., Wang Q., Xie Z.W., Zhou L., Li S.W., Hui F., Wang X.Q., Li B.G., Ma Y.T. (2020). Estimation of maize plant height and leaf area index dynamics using an unmanned aerial vehicle with oblique and nadir photography. Ann. Bot..

[25] Nguy-Robertson A., Gitelson A., Peng Y., Viña A., Arkebauer T., Rundquist D. (2012). Green Leaf Area Index Estimation in Maize and Soybean: Combining Vegetation Indices to Achieve Maximal Sensitivity. Agron. J..

[26] He J.Y., Zhang N., Su X., Lu J.S., Yao X., Cheng T., Zhu Y., Cao W.X., Tian Y.C. (2019). Estimating Leaf Area Index with a New Vegetation Index Considering the Influence of Rice Panicles. Remote Sens..

[27] Zha H.N., Miao Y.X., Wang T.T., Li Y., Zhang J., Sun W.C., Feng Z.Q., Kusnierek K. (2020). Improving Unmanned Aerial Vehicle Remote Sensing-Based Rice Nitrogen Nutrition Index Prediction with Machine Learning. Remote Sens..

[28] Yang Q., Shi L.S., Han J.Y., Chen Z.W., Yu J. (2022). A VI-based phenology adaptation approach for rice crop monitoring using UAV multispectral images. Field Crops Res..

[29] Li J.P., Li J.X., Zhao D.X., Cao Q., Yu F.H., Cao Y.L., Feng S., Xu T.Y. (2025). High-throughput method for improving rice AGB estimation based on UAV multi-source remote sensing image feature fusion and ensemble learning. Front. Plant Sci..

[30] Zhou X., Zheng H.B., Xu X.Q., He J.Y., Ge X.K., Yao X., Cheng T., Zhu Y., Cao W.X., Tian Y.C. (2017). Predicting grain yield in rice using multi-temporal vegetation indices from UAV-based multispectral and digital imagery. ISPRS J. Photogramm. Remote Sens..

[31] Liu Y., Fan K.J., Meng L., Nie C.W., Liu Y.D., Cheng M.H., Song Y., Jin X.L. (2025). Synergistic use of stay-green traits and UAV multispectral information in improving maize yield estimation with the random forest regression algorithm. Comput. Electron. Agric..

[32] Kelsey K.C., Neff J.C. (2014). Estimates of Aboveground Biomass from Texture Analysis of Landsat Imagery. Remote Sens..

[33] Adeluyi O., Harris A., Foster T., Clay G.D. (2022). Exploiting centimetre resolution of drone-mounted sensors for estimating mid-late season above ground biomass in rice. Eur. J. Agron..

[34] Wang Z.L., Ma Y.M., Chen P., Yang Y.G., Fu H., Yang F., Raza M.A., Guo C.C., Shu C.H., Sun Y.J. (2022). Estimation of Rice Aboveground Biomass by Combining Canopy Spectral Reflectance and Unmanned Aerial Vehicle-Based Red Green Blue Imagery Data. Front. Plant Sci..

[35] Li H., Yan X.B., Su P.Y., Su Y.M., Li J.F., Xu Z.X., Gao C.R., Zhao Y., Feng M.C., Shafiq F. (2025). Estimation of winter wheat LAI based on color indices and texture features of RGB images taken by UAV. J. Sci. Food Agric..

[36] Bruce L.M. (2002). Dimensionality reduction of hyperspectral data using discrete wavelet transform feature extraction. IEEE Trans. Geosci. Remote Sens..

[37] Yue J.B., Zhou C.Q., Guo W., Feng H.K., Xu K.J. (2021). Estimation of winter-wheat above-ground biomass using the wavelet analysis of unmanned aerial vehicle-based digital images and hyperspectral crop canopy images. Int. J. Remote Sens..

[38] Liu Y., Feng H.K., Yue J.B., Jin X.L., Fan Y.G., Chen R.Q., Bian M.B., Ma Y.P., Song X.Y., Yang G.J. (2023). Improved potato AGB estimates based on UAV RGB and hyperspectral images. Comput. Electron. Agric..

[39] Stefenon S.F., Seman L.O., da Silva E.C., Finardi E.C., Coelho L.D., Mariani V.C. (2024). Hypertuned wavelet convolutional neural network with long short-term memory for time series forecasting in hydroelectric power plants. Energy.

[40] Yang B.H., Wang M.X., Sha Z.X., Wang B., Chen J.L., Yao X., Cheng T., Cao W.X., Zhu Y. (2019). Evaluation of Aboveground Nitrogen Content of Winter Wheat Using Digital Imagery of Unmanned Aerial Vehicles. Sensors.

[41] Han W.Q., Guan J.Y., Zheng J.H., Liu Y.J., Ju X.F., Liu L., Li J.H., Mao X.R., Li C.R. (2023). Probabilistic assessment of drought stress vulnerability in grasslands of Xinjiang, China. Front. Plant Sci..

[42] Hussain M.H., Abuhani D.A., Khan J., ElMohandes M., Zualkernan I., Ali T. (2023). A Light-Weight Cropland Mapping Model Using Satellite Imagery. Sensors.

[43] Sandonís-Pozo L., Oger B., Tisseyre B., Llorens J., Escolà A., Pascual M., Martínez-Casasnovas J.A. (2024). Leafiness-LiDAR index and NDVI for identification of temporal patterns in super-intensive almond orchards as response to different management strategies. Eur. J. Agron..

[44] Mutanga O., Adam E., Cho M.A. (2012). High density biomass estimation for wetland vegetation using WorldView-2 imagery and random forest regression algorithm. Int. J. Appl. Earth Obs. Geoinf..

[45] Li Y.F., Li C.C., Cheng Q., Duan F.Y., Zhai W.G., Li Z.P., Mao B.H., Ding F., Kuang X.H., Chen Z. (2024). Estimating Maize Crop Height and Aboveground Biomass Using Multi-Source Unmanned Aerial Vehicle Remote Sensing and Optuna-Optimized Ensemble Learning Algorithms. Remote Sens..

[46] da Silva E.C., Finardi E.C., Stefenon S.F. (2024). Enhancing hydroelectric inflow prediction in the Brazilian power system: Comparative analysis of machine learning models and hyperparameter optimization for decision support. Electr. Power Syst. Res..

[47] Hlatshwayo S.T., Mutanga O., Lottering R.T., Kiala Z., Ismail R. (2019). Mapping forest aboveground biomass in the reforested Buffelsdraai landfill site using texture combinations computed from SPOT-6 pan-sharpened imagery. Int. J. Appl. Earth Obs. Geoinf..

[48] Miller T. (2019). Explanation in artificial intelligence: Insights from the social sciences. Artif. Intell..

[49] Li Z.Q. (2022). Extracting spatial effects from machine learning model using local interpretation method: An example of SHAP and XGBoost. Comput. Environ. Urban Syst..

[50] Zhang S.H., He L., Duan J.Z., Zang S.L., Yang T.C., Schulthess U.R.S., Guo T.C., Wang C.Y., Feng W. (2024). Aboveground wheat biomass estimation from a low-altitude UAV platform based on multimodal remote sensing data fusion with the introduction of terrain factors. Precis. Agric..

[51] Liu Y., Feng H.K., Yue J.B., Fan Y.G., Bian M.B., Ma Y.P., Jin X.L., Song X.Y., Yang G.J. (2023). Estimating potato above-ground biomass by using integrated unmanned aerial system-based optical, structural, and textural canopy measurements. Comput. Electron. Agric..

[52] Liang Y.Y., Kou W.L., Lai H.Y., Wang J., Wang Q.H., Xu W.H., Wang H., Lu N. (2022). Improved estimation of aboveground biomass in rubber plantations by fusing spectral and textural information from UAV-based RGB imagery. Ecol. Indic..

[53] Cheng T., Song R.Z., Li D., Zhou K., Zheng H.B., Yao X., Tian Y.C., Cao W.X., Zhu Y. (2017). Spectroscopic Estimation of Biomass in Canopy Components of Paddy Rice Using Dry Matter and Chlorophyll Indices. Remote Sens..

[54] Wang X.K., Xu G.L., Feng Y.H., Peng J.F., Gao Y.Q., Li J., Han Z.L., Luo Q.X., Ren H.J., You X.X. (2023). Estimation Model of Rice Aboveground Dry Biomass Based on the Machine Learning and Hyperspectral Characteristic Parameters of the Canopy. Agronomy.

[55] Fan Y.G., Feng H.K., Yue J.B., Jin X.L., Liu Y., Chen R.Q., Bian M.B., Ma Y.P., Song X.Y., Yang G.J. (2023). Using an optimized texture index to monitor the nitrogen content of potato plants over multiple growth stages. Comput. Electron. Agric..

[56] Zheng H.B., Cheng T., Zhou M., Li D., Yao X., Tian Y.C., Cao W.X., Zhu Y. (2019). Improved estimation of rice aboveground biomass combining textural and spectral analysis of UAV imagery. Precis. Agric..

[57] Dhakal R., Maimaitijiang M., Chang J., Caffe M. (2023). Utilizing Spectral, Structural and Textural Features for Estimating Oat Above-Ground Biomass Using UAV-Based Multispectral Data and Machine Learning. Sensors.

[58] Shu M.Y., Fei S.P., Zhang B.Y., Yang X.H., Guo Y., Li B.G., Ma Y.T. (2022). Application of UAV Multisensor Data and Ensemble Approach for High-Throughput Estimation of Maize Phenotyping Traits. Plant Phenomics.

[59] Yue J.B., Yang G.J., Tian Q.J., Feng H.K., Xu K.J., Zhou C.Q. (2019). Estimate of winter-wheat above-ground biomass based on UAV ultrahigh-ground-resolution image textures and vegetation indices. ISPRS J. Photogramm. Remote Sens..

[60] Xu T.Y., Wang F.M., Shi Z., Miao Y.X. (2024). Multi-scale monitoring of rice aboveground biomass by combining spectral and textural information from UAV hyperspectral images. Int. J. Appl. Earth Obs. Geoinf..

[61] Wu B., Fan L.Q., Xu B.W., Yang J.J., Zhao R.M., Wang Q., Ai X.T., Zhao H.X., Yang Z.R. (2025). UAV-based LiDAR and multispectral sensors fusion for cotton yield estimation: Plant height and leaf chlorophyll content as a bridge linking remote sensing data to yield. Ind. Crops Prod..

[62] Li S.Y., Yuan F., Ata-UI-Karim S.T., Zheng H.B., Cheng T., Liu X.J., Tian Y.C., Zhu Y., Cao W.X., Cao Q. (2019). Combining Color Indices and Textures of UAV-Based Digital Imagery for Rice LAI Estimation. Remote Sens..

[63] Randelovic P., Dordevic V., Miladinovic J., Prodanovic S., Ceran M., Vollmann J. (2023). High-throughput phenotyping for non-destructive estimation of soybean fresh biomass using a machine learning model and temporal UAV data. Plant Methods.

[64] Niu C., Tan K., Jia X.P., Wang X. (2021). Deep learning based regression for optically inactive inland water quality parameter estimation using airborne hyperspectral imagery. Environ. Pollut..

[65] Huete A., Didan K., Miura T., Rodriguez E.P., Gao X., Ferreira L.G. (2002). Overview of the radiometric and biophysical performance of the MODIS vegetation indices. Remote Sens. Environ..

[66] Candiago S., Remondino F., De Giglio M., Dubbini M., Gattelli M. (2015). Evaluating Multispectral Images and Vegetation Indices for Precision Farming Applications from UAV Images. Remote Sens..

[67] Tian Y.C., Yao X., Yang J., Cao W.X., Hannaway D.B., Zhu Y. (2011). Assessing newly developed and published vegetation indices for estimating rice leaf nitrogen concentration with ground- and space-based hyperspectral reflectance. Field Crops Res..

[68] Xiao Y.F., Zhao W.J., Zhou D.M., Gong H.L. (2014). Sensitivity Analysis of Vegetation Reflectance to Biochemical and Biophysical Variables at Leaf, Canopy, and Regional Scales. IEEE Trans. Geosci. Remote Sens..

[69] Daughtry C.S.T., Walthall C.L., Kim M.S., Colstoun E.B.d., McMurtrey J.E. (2000). Estimating Corn Leaf Chlorophyll Concentration from Leaf and Canopy Reflectance. Remote Sens. Environ..

[70] Sandham L.A., Zietsman H.L. (2017). Surface Temperature Measurement from Space: A Case Study in the South Western Cape of South Africa. S. Afr. J. Enol. Vitic..

[71] Haboudane D., Miller J.R., Pattey E., Zarco-Tejada P.J., Strachan I.B. (2003). Hyperspectral vegetation indices and novel algorithms for predicting green LAI of crop canopies: Modeling and validation in the context of precision agriculture. Remote Sens. Environ..

[72] Zhu Y., Yao X., Tian Y., Liu X., Cao W. (2008). Analysis of common canopy vegetation indices for indicating leaf nitrogen accumulations in wheat and rice. Int. J. Appl. Earth Obs. Geoinf..

[73] Broge N.H., Mortensen J.V. (2002). Deriving green crop area index and canopy chlorophyll density of winter wheat from spectral reflectance data. Remote Sens. Environ..

[74] Gitelson A.A. (2004). Wide Dynamic Range Vegetation Index for Remote Quantification of Biophysical Characteristics of Vegetation. J. Plant Physiol..

[75] Motohka T., Nasahara K.N., Oguma H., Tsuchida S. (2010). Applicability of Green-Red Vegetation Index for Remote Sensing of Vegetation Phenology. Remote Sens..

[76] Xue J.R., Su B.F. (2017). Significant Remote Sensing Vegetation Indices: A Review of Developments and Applications. J. Sens..

[77] Jimenez R.B., Lane K.J., Hutyra L.R., Fabian M.P. (2022). Spatial resolution of Normalized Difference Vegetation Index and greenness exposure misclassification in an urban cohort. J. Expo. Sci. Environ. Epidemiol..

[78] Schneider P., Roberts D.A., Kyriakidis P.C. (2007). A VARI-based relative greenness from MODIS data for computing the Fire Potential Index. Remote Sens. Environ..

[79] Cao Q., Miao Y.X., Wang H.Y., Huang S.Y., Cheng S.S., Khosla R., Jiang R.F. (2013). Non-destructive estimation of rice plant nitrogen status with Crop Circle multispectral active canopy sensor. Field Crops Res..

[80] Dash J., Curran P.J., Tallis M.J., Llewellyn G.M., Taylor G., Snoeij P. (2010). Validating the MERIS Terrestrial Chlorophyll Index (MTCI) with ground chlorophyll content data at MERIS spatial resolution. Int. J. Remote Sens..

[81] Lu J.J., Miao Y.X., Shi W., Li J.X., Yuan F. (2017). Evaluating different approaches to non-destructive nitrogen status diagnosis of rice using portable RapidSCAN active canopy sensor. Sci. Rep..

[82] Haboudane D., Tremblay N., Miller J.R., Vigneault P. (2008). Remote Estimation of Crop Chlorophyll Content Using Spectral Indices Derived From Hyperspectral Data. IEEE Trans. Geosci. Remote Sens..

[83] Broge N.H., Leblanc E. (2001). Comparing prediction power and stability of broadband and hyperspectral vegetation indices for estimation of green leaf area index and canopy chlorophyll density. Remote Sens. Environ..

