# Supplementary material for: Improved Multi-Stage Rice Above-Ground Biomass Estimation Using Wavelet-Texture-Fused Vegetation Indices from UAV Remote Sensing
Source: Plants (Basel). 2025 Sep 18;14(18):2903. doi: 10.3390/plants14182903 (PMC12473360; doi:10.3390/plants14182903)
Supplement: Supplementary file 1 [file plants-14-02903-s001.zip › plants-3854740-supplementary.pdf]

# Supplemental information for

# Improved Multi-Stage Rice Above-Ground Biomass Estimation Using Wavelet Texture Fused Vegetation Indices from UAV Remote Sensing

**Jinpeng Li [1,2], Qiang Cao [1,2], Shuaipeng Wang [1,2], Jiayi Li [1,2], Dongxue Zhao [1,2,3], Shuai Feng [1,2,3], Yingli Cao [1,2,3] and Tongyu Xu [1,2,3,*]**

[1] College of Information and Electrical Engineering, Shenyang Agricultural University, Shenyang 110866, China

[2] National Digital Agriculture Sub-center of Innovation (Northeast Region), Shenyang 110866, China

[3] Key Laboratory of Intelligent Agriculture in Liaoning Province, Shenyang 110866, China

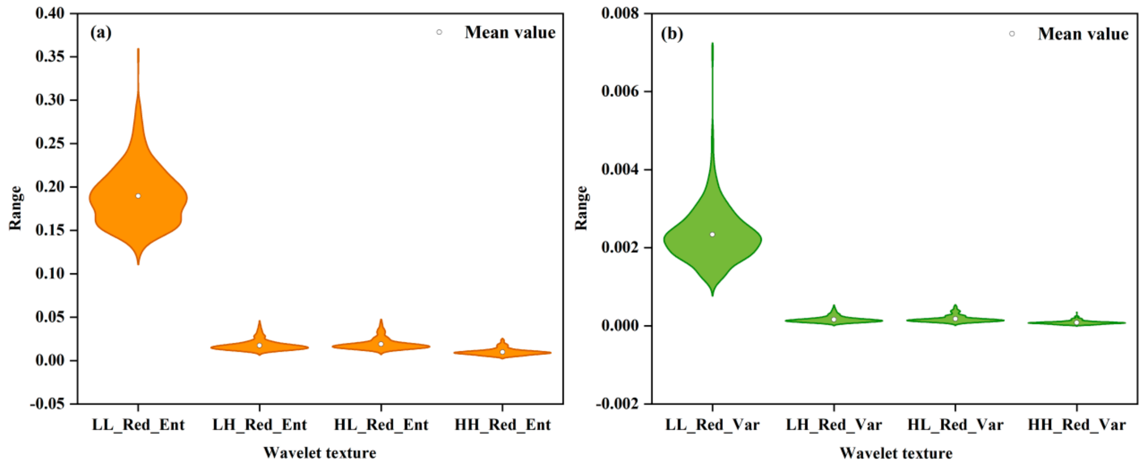


Figure S1. Statistical distribution of high-frequency and low-frequency texture features (using variance and entropy of multispectral red band images as examples)

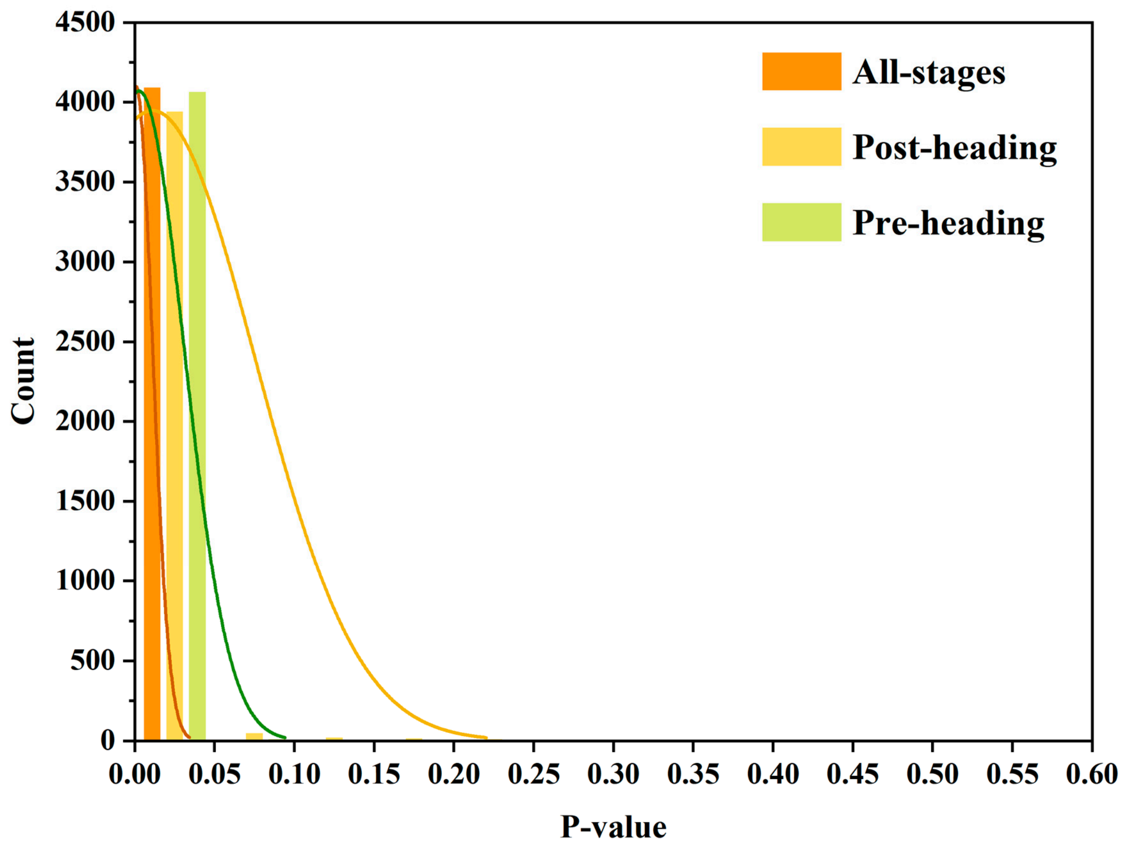


Figure S2. Statistical results of normality tests for remote sensing variables at different growth stages of rice and AGB.

Table S1. 21 VIs used in this study.

| Index name | Formula | Reference |
|---|---|---|
| NDVI (Normalized difference vegetation index) | (NIR-R)/(NIR+R) | [65] |
| GNDVI (Green normalized difference vegetation index) | (NIR-G)/(NIR+G) | [66] |
| NDRE (Normalized difference red edge index) | (NIR-RE)/(NIR+RE) | [67] |
| LCI (Leaf chlorophyll index) | (NIR-RE)/(NIR+R) | [68] |
| OSAVI (Optimized soil adjusted vegetation index) | (NIR-R)/(NIR+R+0.16) | [69] |
| SRI (Simple ratio index) | NIR/R | [70] |
| MSRI (Modified simple ratio index) | (NIR/R-1)/ Sqr (NIR/R)+1 | [71] |
| DVI (Difference vegetation index) | NIR-R | [72] |
| RVI (Ratio vegetation index) | R/NIR | [73] |
| WDRVI (Wide dynamic range vegetation index) | (0.2*NIR-R)/(0.2*NIR+R) | [74] |
| GRVI (Green ratio vegetation index) | NIR/G | [75] |
| TDVI (Transformed difference vegetation index) | $(0.5+(NIR-R))/(NIR+R)^{0.5}$ | [76] |
| SAVI (Soil adjusted vegetation index) | (NIR-R)*(1+0.5)/(NIR+R+0.5) | [71] |
| EVI (Enhanced vegetation index) | 2.5*(NIR-R)/(NIR+2.4*R+1) | [65] |
| NGRVI (Normalized green red vegetation index) | (G-R)/(G+R) | [77] |
| VREI (Vogelmann red edge index) | NIR/RE | [78] |
| GRDVI (Green re-normalized different vegetation index | (NIR-G)/Sqr(NIR+G) | [79] |
| MTCI (Meris terrestrial chlorophyll index) | (NIR-RE)/(RE-R) | [80] |
| RETVI (Red edge transformed vegetation index) | 0.5*(120*(NIR-R)-200*(RE-R)) | [81] |

| | | |
|---|---|---|
| TCI (Triangular chlorophyll index) | 1.2*(RE-G)-1.5*(R-G)*Sqr(RE/R) | [82] |
| TVI (Triangular vegetation index) | 0.5*(120*(NIR-G)-200*(R-G)) | [83] |

Table S2. Summary of hyperparameters for the RF models.

| Hyperparameters | Space | Description |
|---|---|---|
| n_estimators | 100-1000 | Number of decision trees |
| max_depth | 2-50 | Maximum depth of the decision tree |
| min_samples_split | 2-50 | The minimum number of samples a node must possess before continuing to split |
| min_samples_leaf | 1-50 | Minimum number of samples per leaf node |
| max_features | sqrt/log2/None | Maximum number of features considered for division |

## References


65. Huete, A.; Didan, K.; Miura, T.; Rodriguez, E.P.; Gao, X.; Ferreira, L.G. Overview of the radiometric and biophysical performance of the MODIS vegetation indices. Remote Sensing of Environment. 2002, 83, 195-213.

66. Candiago, S.; Remondino, F.; De Giglio, M.; Dubbini, M.; Gattelli, M. Evaluating Multispectral Images and Vegetation Indices for Precision Farming Applications from UAV Images. Remote Sensing. 2015, 7, 4026-4047.

67. Tian, Y.C.; Yao, X.; Yang, J.; Cao, W.X.; Hannaway, D.B.; Zhu, Y. Assessing newly developed and published vegetation indices for estimating rice leaf nitrogen concentration with ground- and space-based hyperspectral reflectance. Field Crops Research. 2011, 120, 299-310.

68. Xiao, Y.F.; Zhao, W.J.; Zhou, D.M.; Gong, H.L. Sensitivity Analysis of Vegetation Reflectance to Biochemical and Biophysical Variables at Leaf, Canopy, and Regional Scales. Ieee Transactions on Geoscience and Remote Sensing. 2014, 52, 4014-4024.

69. Daughtry, C.S.T.; Walthall, C.L.; Kim, M.S.; Colstoun, E.B.d.; McMurtrey, J.E. Estimating Corn Leaf Chlorophyll Concentration from Leaf and Canopy Reflectance. Remote Sensing of Environment. 2000, 74, 229-239.

70. Sandham, L.A.; Zietsman, H.L. Surface Temperature Measurement from Space: A Case Study in the South Western Cape of South Africa. South African Journal of Enology and Viticulture. 2017, 18.

71. Haboudane, D.; Miller, J.R.; Pattey, E.; Zarco-Tejada, P.J.; Strachan, I.B. Hyperspectral vegetation indices and novel algorithms for predicting green LAI of crop canopies: Modeling and validation in the context of precision agriculture. Remote Sensing of Environment. 2003, 90, 337-352.

72. Zhu, Y.; Yao, X.; Tian, Y.; Liu, X.; Cao, W. Analysis of common canopy vegetation indices for indicating leaf nitrogen accumulations in wheat and rice. International journal of applied earth observation and geoinformation. 2008, 10, 1-10.

73. Broge, N.H.; Mortensen, J.V. Deriving green crop area index and canopy chlorophyll density of winter wheat from spectral reflectance data. Remote Sensing of Environment. 2002, 81, 45-57.

74. Gitelson, A.A. Wide Dynamic Range Vegetation Index for Remote Quantification of Biophysical Characteristics of Vegetation. Journal of Plant Physiology. 2004, 161, 165-173.

75. Motohka, T.; Nasahara, K.N.; Oguma, H.; Tsuchida, S. Applicability of Green-Red Vegetation Index for Remote Sensing of Vegetation Phenology. Remote Sensing. 2010, 2, 2369-2387.

76. Xue, J.R.; Su, B.F. Significant Remote Sensing Vegetation Indices: A Review of Developments and Applications. Journal of Sensors. 2017, 2017, 1353691.

77. Jimenez, R.B.; Lane, K.J.; Hutyra, L.R.; Fabian, M.P. Spatial resolution of Normalized Difference Vegetation Index and greenness exposure misclassification in an urban cohort. Journal of Exposure Science and Environmental Epidemiology. 2022, 32, 213-222.

78. Schneider, P.; Roberts, D.A.; Kyriakidis, P.C. A VARI-based relative greenness from MODIS data for computing the Fire Potential Index. Remote Sensing of Environment. 2007, 112, 1151-1167.

79. Cao, Q.; Miao, Y.X.; Wang, H.Y.; Huang, S.Y.; Cheng, S.S.; Khosla, R.; Jiang, R.F. Non-destructive estimation of rice plant nitrogen status with Crop Circle multispectral active canopy sensor. Field Crops Research. 2013, 154, 133-144.

80. Dash, J.; Curran, P.J.; Tallis, M.J.; Llewellyn, G.M.; Taylor, G.; Snoeij, P. Validating the MERIS Terrestrial Chlorophyll Index (MTCI) with ground chlorophyll content data at MERIS spatial resolution. International Journal of Remote Sensing. 2010, 31, 5513-5532.

81. Lu, J.J.; Miao, Y.X.; Shi, W.; Li, J.X.; Yuan, F. Evaluating different approaches to non-destructive nitrogen status diagnosis of rice using portable RapidSCAN active canopy sensor. Scientific Reports. 2017, 7, 14073.

82. D., H.; N., T.; R., M.J.; P., V. Remote Estimation of Crop Chlorophyll Content Using Spectral Indices Derived From Hyperspectral Data. IEEE Transactions on Geoscience and Remote Sensing. 2008, 46, 423-437.

83. Broge, N.H.; Leblanc, E. Comparing prediction power and stability of broadband and hyperspectral vegetation indices for estimation of green leaf area index and canopy chlorophyll density. Remote Sensing of Environment. 2001, 76, 156-172.
